# IL-6 of follicular fluid and outcome of *in vitro* fertilization

**DOI:** 10.1097/MD.0000000000029624

**Published:** 2022-07-22

**Authors:** Aleksandra Z. Stojanovic Gavrilovic, Jelena M. Cekovic, Aida Z. Parandilovic, Aleksandar B. Nikolov, Predrag S. Sazdanovic, Aleksandra M. Velickovic, Marija V. Andjelkovic, Marija P. Sorak

**Affiliations:** a Clinical Center Kragujevac, Clinic of Gynecology and Obstetrics, Department of Biomedically Assisted Fertilization, Kragujevac, Serbia; b University of Kragujevac, Faculty of Medical Sciences, PhD student, Kragujevac, Serbia; c University of Kragujevac, Faculty of Medical Sciences, Department of Anatomy, Kragujevac, Serbia; d Clinical Center Kragujevac, Department of Laboratory Diagnostics, Kragujevac, Serbia; e University of Kragujevac, Faculty of Medical Sciences, Department of Biochemistry, Kragujevac, Serbia; f University of Kragujevac, Faculty of Medical Sciences, Department of Gynecology and Obstetrics, Kragujevac, Serbia.

**Keywords:** biomedically assisted fertilization, follicular fluid, IVF, oocytes, IL-6

## Abstract

The quality of an oocyte is influenced by its microenvironment, which includes cumulus cells and follicular fluid, as well as cells of the immune system and their products. The ovarian interleukins, which are secreted by the granulosa cells and other immune cells within the ovaries and follicles, regulate various functions between the cells. IL-6 is a cytokine that is present in the follicular fluid and may affect the quality of oocytes. There are some inconsistencies in the literature regarding the concentration of interleukin 6 in the follicular fluid.

The main objective of this study was to examine whether the concentration of interleukin 6 in the follicular fluid affects the outcome of IVF.

This study involved 83 patients who underwent IVF. Follicular fluid was used as the biological material for the analysis. Examination of the obtained follicular fluid and collection of oocytes under a stereomicroscope was performed in the embryological laboratory. The concentration of IL-6 in the follicular fluid was analyzed. IVF and ICSI methods were used as the fertilization methods. Pregnancy was confirmed by the positive serum β-hCG level. The software package SPSS 20 was used for statistical data processing.

Analysis of the follicular fluid samples showed a correlation between the concentration of IL-6 in the follicular fluid and the outcome of IVF. The concentration of IL-6 in the follicular fluid was higher in patients with confirmed pregnancy (9.55 ± 7.47 ng/ml).

Based on our results, we conclude that the concentration of IL-6 affects the outcome of IVF. If the range of IL-6 concentration is between 3,67 ng/ml and 10 ng/ml, we can expect good IVF outcome with vital pregnancy.

## 1. Introduction

Cytokines were originally identified as products of immune system cells and are important immune response mediators. Cytokines can stimulate or inhibit cell growth, regulate cell differentiation, induce cell chemotaxis, and modulate the expression of other cytokines.^[1]^ Cytokines have been found to regulate the reproductive process by influencing the immune environment within the follicle itself, as well as the uterus.^[2]^

Cytokines consist of smaller water-soluble proteins and glycoproteins. They are classified into lymphokines, interleukins, and chemokines based on their function, cell origin, and target cells.^[3]^ Cytokines can modulate the function of immune cells within the ovary in the context of reproduction.^[3]^

The ovarian interleukins, which are secreted by the granulosa cells and other immune cells within the ovaries and follicles along with hormonal changes, regulate various functions, including folliculogenesis, oogenesis, ovulation, fertilization, embryonic development, implantation, formation, and regression of the corpus luteum.^[4]^ One of the success factors of in vitro fertilization (IVF) is the quality of the obtained oocytes. The quality of the oocyte is significantly affected by the environment in which it is located or the so-called microenvironment. Defining certain parameters of the microenvironment, which can be easily and quickly detected, which enable the differentiation of oocytes that are of better or worse quality, could potentially increase the success of IVF. In the follicles, oocytes undergo growth and maturation. The follicular wall consists of granulosa and theca cells, which are separated by the basal membrane. The maturation process is carried out through several stages within follicles. During follicular growth, its interior is filled with follicular fluid that is made by the filtration of the blood plasma constituents and by the secretory activity of granulosa and theca cells.^[3]^ One of the normal constituents of follicular fluid is interleukin 6 (IL-6).^[5]^

IL-6 is a pleiotropic cytokine with multiple effects that can vary depending on the physiological environment. IL-6 promotes T cell population expansion, B cell differentiation, and proliferation of many cells it can also regulate the acute phase response and various homeostasis functions, including glucose metabolism, lipid metabolism, and insulin resistance. IL-6 also affects the endocrine and nervous systems. It is also involved in the occurrence and development of various cancers.^[6,7]^ IL-6 also plays an important role in follicle development. Regarding folliculogenesis, cytokines regulate cell proliferation or differentiation, follicle survival or atresia, and oocyte maturation.^[8]^ IL-6 levels in the follicular fluid are significantly higher than those in the serum.^[9,10]^ In the polycystic ovary syndrome, IL-6 levels increase in the serum as well as in the follicular fluid due to an increased secretion by the granulosa cells, and some studies have shown that increased IL-6 levels may be associated with hyperandrogenism and insulin resistance.^[11]^

Clinical studies have been conducted to investigate the potential role of IL-6 in human oocyte maturation and embryo development; however, to date, the answers have not been definitive. Some studies have shown that high levels of IL-6 in follicular fluid have a positive effect on oocyte maturation.^[12,13]^ High levels of IL-6 in embryo culture are associated with an increased rate of clinical pregnancies and implantation of embryos.^[10,14–16]^ However, other studies have reported contradictory results. Higher levels of IL-6 correlate with poor embryo quality, and patients are less likely to become pregnant.^[17–19]^ It has been concluded that IL-6 inhibits oocyte development.^[20]^ In addition, some studies have suggested that IL-6 does not affect the clinical rate of pregnancy, as well as the quality of oocytes.^[21–23]^

This study examined the level of IL-6 in follicular fluid from the stimulated cycles. The main objective was to examine whether the concentration of IL-6 in the follicular fluid affects the quality of oocytes and embryos, and therefore, the outcome of the process of in vitro fertilization.

## 2. Material and Methods

### 2.1. Study population

The study involved 83 women who were included in the National Program for Biomedically Assisted Fertilization funded by the Ministry of Health of the Republic of Serbia, and who decided, during the commission process, to subject themselves to the process of biomedically assisted fertilization at the Department of Biomedically Assisted Fertilization, at the Clinic of Gynecology and Obstetrics, of the Clinical Center Kragujevac. The study was conducted in the period from September 2020 to Jun 2021 and approved by the Ethics Committee of the Clinical Center Kragujevac (decision number 01-20-582/ 25.06.2020.). All 83 participants of the study, prior to being involved in the study, were informed about the research objectives, and all of them signed the consent to participate in the study. The examined patients were divided into 2 groups based on the outcome of in vitro fertilization: the first group consisted of patients in whom pregnancy was not confirmed, while the second group consisted of patients in whom pregnancy was confirmed. Patients were also divided into 2 groups according to age: the first group was 35 years old and younger than 35 years, while the second group included those older than 35 years.

*The inclusion criteria were as follows:* spouses, that is, extramarital partners who have exhausted other possibilities of infertility treatment, spouses or extramarital partners who have 1 child in the existing union obtained in the IVF procedure, preserved ovarian function, and normal body mass index (BMI). Regular findings of the following microbiological and virological analyses: vaginal and cervical smear for bacteria, fungi and Chlamydia, bacterial vaginosis, Toxoplasma gondii, Rubella, syphilis, hepatitis B (HbsAg), hepatitis C virus (HCV), human immunodeficiency virus (HIV), hormonal status (the results obtained on the second or third day of the menstrual cycle), f*ollicle-stimulating hormone* (FSH), luteinizing hormone (LH), estradiol, progesterone, testosterone, prolactin, thyroid-stimulating hormone (TSH), triiodothyronine (FT3), thyroxine (FT4), antiMullerian hormone (AMH), and cervical screening results (Pap smear, colposcopy), ultrasound examination performed with a vaginal probe, examination of the fallopian tube patency by hysterosalpingography.

*The criteria for exclusion from the study were* as follows: couples in whom other options for infertility treatment have not been exhausted, women in whom the ovarian reserve is not preserved, women with BMI > 30 kg/m², anomalies and benign tumors of the uterus, fallopian tubes, and ovaries that prevent the process of IVF, the onset and development of pregnancy, the existence of malignant or suspected tumors of the uterus, fallopian tubes, and ovaries; any diseases (cardiovascular diseases, immunological, infectious diseases, neurological or psychiatric diseases) if they are without permission of the appropriate specialist to perform the IVF procedure, diseases in which anesthesia or pregnancy could potentially endanger the patient’s life. Patients who suffer from any other endocrine disease that has been confirmed to affect fertility were excluded from the study.

### 2.2. Material

During the process of ovarian stimulation, several therapeutic agents were used such as: urinary gonadotropins (Menopur 75 i.j., menotrophin-human menopausal gonadotropin, Ferring Pharmaceuticals, Germany; Merional 75 ij, menotrophin-human menopausal gonadotropin, IBSA Institut Biochimique SA, Switzerland), recombinant gonadotropins (Gonal- F 75 i.j., Follitropin alfa, Merck Serono, Modugno, Italy) and GnRh antagonists (Cetrotide 0.25 mg/ml, Cetrorelix acetate, Merck Healthcare KGaA, Germany). Final oocyte maturation was induced with either 5000IU chorionic gonadotropin (Pregnyl, NV Organon, The Netherlands) or 250 µg chorionic gonadotropin alfa (Ovitrelle, Merck Serono SpA, Italy). In addition, after oocyte aspiration, progesterone depot ampoules (125 mg) (Hydroxyprogesterone caproate, Galenika, Serbia) were used to support the luteal phase.

Oocyte aspiration was performed under ultrasound control using an aspiration pump (K-MAR-5200, Cook® Vacuum Pump, Australia) and a double lumen aspiration needle (Cook® EchoTip® Double Lumen Aspiration Needle, Australia).

Follicular fluid was used as the biological material for the analysis. Oocytes and embryos were evaluated according to the criteria of the Istanbul consensus of clinical embryologists.

The media used for the cultivation of oocytes and embryos were Gamete Buffer, Fertilization Medium, Cleavage Medium, and Blastocyst Medium (Cook Medical Inc., Australia). Hyaluronidase Enzymes (Hyasa, Fertil Pro, Germany), Ovoil, Polyvinylpyrrolidone (PVP), and Follicule Flush buffer (Cook Medical Inc., Australia) were also used as the media. The incubators used for the medium equilibration and for the oocyte and embryo cultivation were K-MINK 1000 incubators (William a COOK Australia Pty Ltd). The plastic disposables used for the oocyte collection as well as for the embryo cultivation were IVF tested and approved by Vitrolife, Gothenburg, Sweden. Embryo transfer was performed in Embryoglue medium (Vitrolife, Gothenburg, Sweden) with an embryo transfer catheter.

Cultivation from gametes to blastocysts took place in K-MINC incubators at a temperature of 37°C in an atmosphere of 6% CO_2_ (carbon dioxide) and at a humidity of 95%.

All plastic dishes and media were sterile and IVF was approved. All media were delivered in the original packaging and used as the finished media.

### 2.3. Methods

Follicular fluid was obtained by puncturing and aspirating follicles > 18 mm after controlled ovarian stimulation in the intervention room at the Department of Biomedically Assisted Fertilization. Examination of the obtained follicular fluid and collection of oocytes under a stereomicroscope were performed under controlled conditions in the embryological laboratory at the Department of Biomedically Assisted Fertilization. The embryological procedures included multiple steps: collection of oocytes, IVF, intracytoplasmatic sperm injection (ICSI), fertilization check, embryo evaluation, and embryo transfer. The biochemical parameters of the follicular fluid were analyzed at the Department of Laboratory Diagnostics of the Clinical Center Kragujevac and the Institute of Public Health in Kragujevac.

### 2.4. Stimulation of ovulation

The basal hormonal status was determined in all patients on the second or third day of the menstrual cycle before they were included in the IVF process. The serum levels of estradiol, progesterone, follicle-stimulating hormone, and luteinizing hormone were determined.

A short stimulation protocol was used depending on the gynecologist assessment based on ultrasound findings and hormonal status.

Ovulation stimulation was initiated on the second day of the menstrual cycle with urinary gonadotropins (Merional or Menopur) and/or recombinant gonadotropins (Gonal-F). The initial dose of gonadotropin depended on the gynecologist assessment based on the woman’s hormonal status, age, ovarian reserves, and the woman’s response to previous stimulations. From the fifth day of the stimulation, the serum estradiol levels were measured daily, and ultrasound examination was performed with a vaginal probe until the stop injection. With the increase of estradiol and in the presence of follicles ≥ 14 mm, the application of a gonadotropin-releasing hormone (GnRH) antagonist (Cetrotide) started.

During stimulation, the growth of follicles and the level of sex hormones in the blood were monitored by ultrasound, with successive ultrasound and laboratory examinations. The stimulation lasted until the leading follicle reached a diameter of 20 mm or 2 or more follicles 18 mm in diameter. When the increase in the concentration of estradiol in the serum fit the presence of 2 or more follicles > 18 mm, choriogonadotropin alfa was applied (Ovitrelle, Merck Serono S. PA, Italy) at a dose of 250 mcg 34–36 hours before follicle aspiration.

### 2.5. Puncture and aspiration of follicles

Puncture and aspiration of follicles is a surgical intervention performed under ultrasound control while using a double-lumen aspiration needle (Cook® EchoTip® Double Lumen Aspiration Needle, Australia) and the aspiration pump K-MAR-5200 (Cook® Vacuum Pump). During this process, the patient was under general anesthesia, and the intervention lasted about 15 to 20 minutes, depending on the number and availability of follicles in the ovaries. After the aspiration of the follicles had been completed, the obtained follicular fluid was examined under a microscope in the embryological laboratory, and the number of oocytes and their quality were determined.

Follicular fluid, in which the oocytes were found, was extracted for laboratory analysis. The entire amount of the follicular fluid was centrifuged at 3000 rpm for 10 minutes to separate the pure follicular fluid without cellular elements. For the analysis, 5 ml of the total amount of follicular fluid was taken.

The levels of interleukin-6 in the follicular fluid were analyzed by a chemiluminescence immunoassay on the UniCel 600 apparatus, Becman Coulter, Department of Laboratory Diagnostics of the Clinical Center Kragujevac.

### 2.6. Embryological procedures during the biomedically assisted fertilization process

The quality of oocytes was assessed after oocyte aspiration. Oocyte quality was first assessed on the basis of the cumulus-corona-oocyte complex. The oocyte goes through 5 stages of maturity: immature (tightly attached cumulus cells and corona next to the ovum, the ooplasm is not clearly visible), almost mature (expanded cumulus, compact corona is located next to the oocyte but not tightly attached), mature (expanded radial corona, visible zone of pellucida and clear ooplasm), postmature (expanded cumulus with dark lumps, irregular and incomplete radial corona, granular and dark ooplasm), and atretic (dark ooplasm, an irregular shape often without cumulus, corona with lumps). After separation of the cumulus cells, the oocyte maturity was estimated on the basis of the nuclear maturity (Table 1).

**Table 1:** Oocyte maturity, embryo quality on the third day after fertilization and blastocyst.

Oocyte maturity	
GV cells (the oocyte at the stage of a germinal vesicle)	The presence of the germinal vesicle in the ooplasm and the absence of a polar body, such oocyte is diploid in prophase I
MI oocytes (the oocytes in metaphase I)	The absence of vesicles in the ooplasm and the absence of the first polar body, such oocyte is also diploid
M II oocytes (the oocyte in metaphase II)	The absence of vesicles in the ooplasm, the presence of the first polar body, such oocyte is haploid and the only one that can undergo fertilization
Embryo quality 3 day after fertilization	
Class A	Excellent, without or with 1–10% fragmentation, perfect symmetry
Class B	Medium, with 11–25% of fragmentation, moderate asymmetry
Class C	Poor, > 25% of fragmentation, expressed asymmetry
Blasocyst quality	
According to the appearance of ICM	A A large number of closely grouped cells
	B A large number of cells that are not closely grouped
	C Very few cells
According to the appearance of the trophectoderm	A Many cells form a cohesive epithelium
	B A small number of cells that form a loose epithelium
	C A very small number of cells

GV = germina vesicle, ICM = inner cell mass.

The oocytes at the germinal vesicle stage or MI stage are not suitable for work, and their maturation must be waited for. An assessment of oocyte maturity can also be established based on cytoplasmic maturity. The presence of dark accumulations in the ooplasm is a sign of cytoplasmic immaturity, even if the oocyte is in the M II phase.

IVF or ICSI was used as the fertilization method. After incubation, 16–20 hours after fertilization, the occurrence of fertilization was checked. Fertilization occurs if 2 pronuclei and 2 polar bodies are present.

For embryo quality assessment, the criteria of the Istanbul consensus of clinical embryologists were used as a reference framework.^[24]^ The criteria included the assessment of the degree of fragmentation and blastomere symmetry on the third day after fertilization (Table 1). On the fifth day after fertilization, embryos were at the blastocyst stage. Blastocyst quality was assessed according to the stage of development (RB: round blastocyst, BL: blastocyst, EB: expanded blastocyst, and HB: hatching blastocyst), according to the appearance of inner cell mass (ICM) (Table 1) and according to the appearance of the trophectoderm (Table 1).

On the third or fifth day after oocyte aspiration, embryo transfer of a maximum of 3 embryos was performed under the control of transabdominal ultrasound. Embryo transfer was performed in EmbryoGlue medium (Vitrolife, Gothenburg, Sweden) with an embryo transfer catheter (Frydman ^®^Ultrasoft Echo, Laboratoire CCD, Paris, France). From the day of oocyte aspiration, the patients received progesterone depot intramuscularly (125 mg or half ampoule per day) to support the luteal phase. Pregnancy was confirmed by a positive serum level of the hormone beta human chorionic gonadotropin (β-hCG) 14 days after embryo transfer, while clinical pregnancies were confirmed by transvaginal ultrasound of a gestational sac with a vital embryo at 6 weeks of gestation.

### 2.7. Statistical data processing

Before statistical data processing, the correctness of the distribution of the obtained values was confirmed using the *Kolmogorov-Smirnov* test. Based on the obtained value of *p* by the *Kolmogorov-Smirnov* test, the test used for the statistical analysis was determined (the parametric test ANOVA for *P* > .05) for samples having a normal distribution or nonparametric *Mann-Whitney*, *Kruskal-Wallis* and *Chi-Square Test* if *P* < .05, for samples that do not have a normal distribution. Spearman rank correlation coefficient was used to determine the direction and strength of the relationships between the 2 variables. The coefficients (*rho*) may have only values from –1 to + 1. The sign indicates whether the correlation is positive (both variables together decrease and increase) or negative (one variable decreases when the other increases and vice versa). The value of this coefficient showed the strength of the bond (0.10 ÷ 0.29 – small; 0.30 ÷ 0.49 – medium; 0.50 ÷ 1.00 –high). The predictive efficiency of the examined parameters was analyzed using ROC analysis. The values of the obtained data were considered statistically significant at *P* < .05. The software package SPSS 20 was used for statistical data processing. All values are expressed as mean values, and are presented in tables and graphs.

## 3. Results

A total of 83 patients were included in this study. The basic characteristics of the patients are summarized in Table 2. The average value for the patients’ age, BMI and AMH concentration in the serum was 36.94 ± 4.54 years, 27.2 ± 1.92 kg/m^2^ and 2.32 ± 2.17 ng/mL. The mean value of the obtained oocytes per woman was 7.71 ± 4.77, while the number of mature oocytes per woman was 4.72 ± 3.03. The ICSI method was used in 65.10% of the cases. The average of 3.93 ± 2.55 embryos per woman was obtained. The clinical pregnancy rate was 5.6%. The mean value of IL-6 concentration in the follicular fluid on the day of oocyte aspiration was 6.58 ± 6.56 ng/mL, the maximum value of IL-6 in the follicular fluid was 44.0 ng/mL and which was considered to be an extremely high value.

**Table 2:** Demographic characteristics of patients.

Parameter		Mean	Mediana	SD	Minimum	Maximum
**Age (yr**)		36.94	37	4.54	27	46
**BMI**		27.2	27	1.92	23	31
**AMH (ng/mL**)		2.32	1.91	2.17	0.10	10.36
**Oocytes retrieved**		7.71	7	4.77	1	20
**MII oocytes**		4.72	4.00	3.03	1	15
**Methods (%**)	IVF	10.80	NA	NA	NA	NA
	ICSI	65.10	NA	NA	NA	NA
	IVF/ ICSI	24.10	NA	NA	NA	NA
**Embryos**		3.93	3	2.55	0	12
**Clinical pregnancy rate (%**)		56.6	NA	NA	NA	NA
**FF IL-6 on oocyte retrieval day (ng/mL**)		6.58	4.90	6.56	1.00	44.00

AMH = antimullerian hormone, BMI = body mass index, FF = folicular fluid, ICSI = Intracytoplasmic sperm injection, IVF = in vitro fertilization, NA = not available, SD = standard deviation.

**Figure 1.**
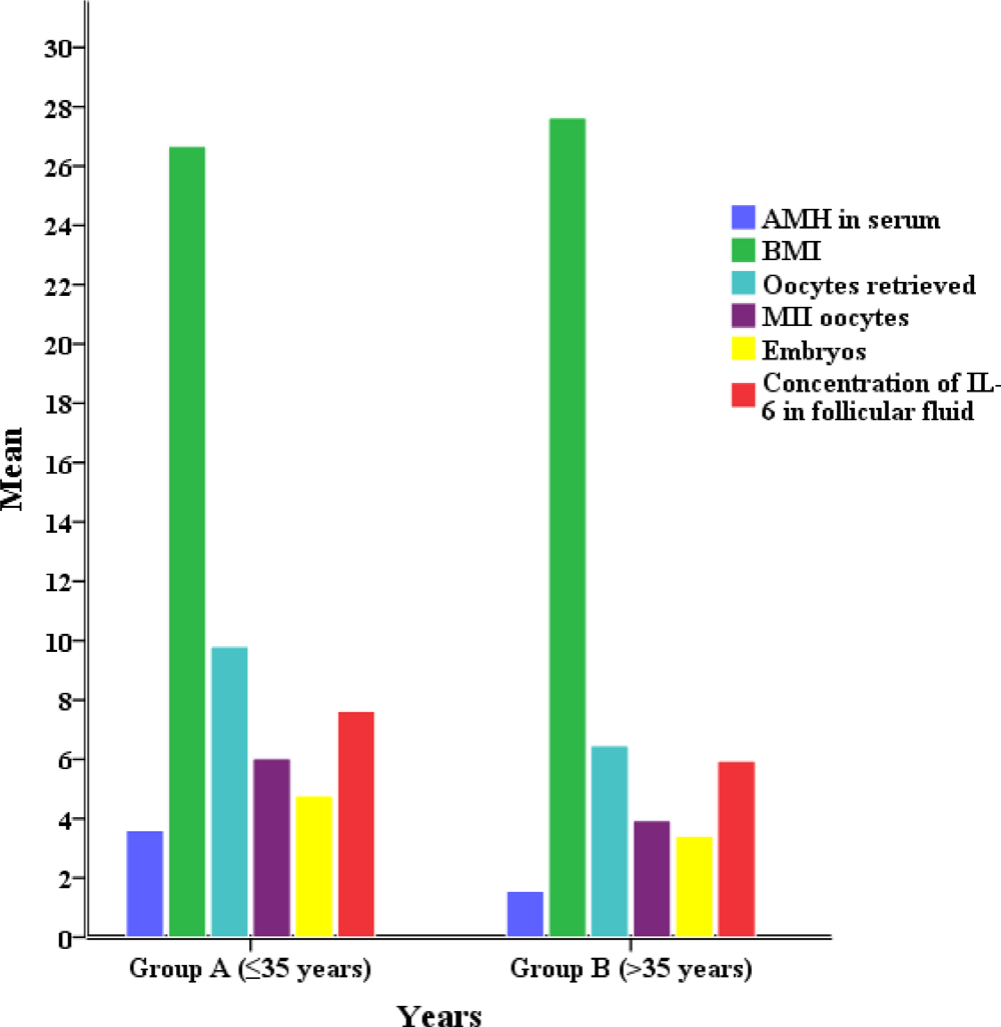
Comparison between clinical characteristics and years.

Table 3 shows the comparison of results for the 2 groups of patients divided by age group (Fig. 1). The first group (group A) included patients aged ≤ 35 years, while the second group (group B) included patients aged > 35 years. A statistically significant difference between 2 groups of patients was confirmed for BMI (*P* = .023), the serum AMH concentration (*P* = .000), the number of oocytes obtained (*P* = .001), the number of mature MII cells (*P* = .001), the number of embryos obtained (*P* = .006), the concentration of IL-6 in the follicular fluid (*P* = .021). There was a statistically significant difference in clinical pregnancy (*P* = .013) between the 2 groups.

**Table 3:** Comparison between clinical characteristics and age.

**Parameter**		Group A (n = 31)≤35 years	Group B (n = 52)>35 years	*P* value
**BMI**		26.68 ± 1.78	27.63 ± 1.92	**0.023** *
**AMH (ng/ mL**)		3.59 ± 2.75	1.55 ± 1.24	**0.000** *
**Oocytes retrieved**		9.81 ± 4.61	6.46 ± 4.54	**0.001** *
**MII oocytes**		6.03 ± 2.89	3.94 ± 2.87	**0.001** *
**Methods (%**)	IVF	19.4	5.8	0.125†
	ICSI	54.8	71.2	
	IVF/ ICSI	25.8	23.1	
**Embryos**		4.77 ± 2.33	3.42 ± 2.56	**0.006** *
**FF IL-6 on oocyte retrieval day (ng/mL**)		7.63 ± 6.46	5.95 ± 6.16	**0.021** *
**Clinical pregnancy rate (%**)	Nonpregnancy	25.8	53.8	**0.013** †
	Pregnancy	74.2	46.2	

The values are given as mean ± standard deviation. Statistically significant *P* values are given in bold.

AMH: antiMullerian hormone; BMI: Body mass index; FF: Follicular fluid concentrations; Group A: ≤35 years; Group B: >35 years; IVF = in vitro fertilization; ICSI: intracytoplasmic sperm injection; IL-6: Interleukin 6.

* *P* value estimated by Mann–Whitney *U* test.

† *P* value estimated by Pearson’s Chi-square test.

Higher serum AMH values (3.59 ± 2.75 ng/mL), a higher number of oocytes obtained (9.81 ± 4.61) and the number of embryos (4.77 ± 2.33) and higher concentration of IL-6 (7.63 ± 6.46 ng/mL) were observed in group A, to which younger patients belonged. In elderly patients, lower values of these parameters were noted: AMH in the serum (1.55 ± 1.24 ng/mL), number of oocytes (6.46 ± 4.54), number of embryos (3.42 ± 2.56), and IL-6 concentration (5.95 ± 6.16 ng/mL). Elderly patients in group B had higher BMI values (27.63 ± 1.92 kg/m^2^).

**Figure 2.**
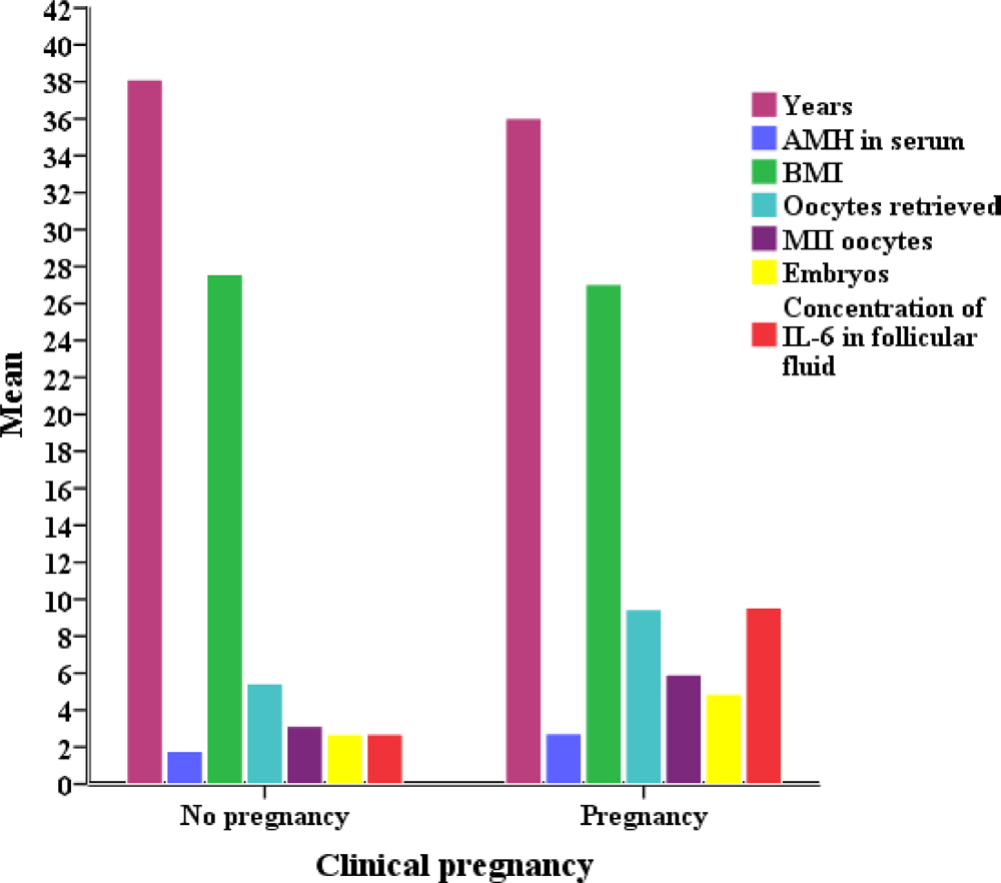
Comparison between nonpregnancy and pregnancy groups for clinical characteristics.

**Figure 3.**
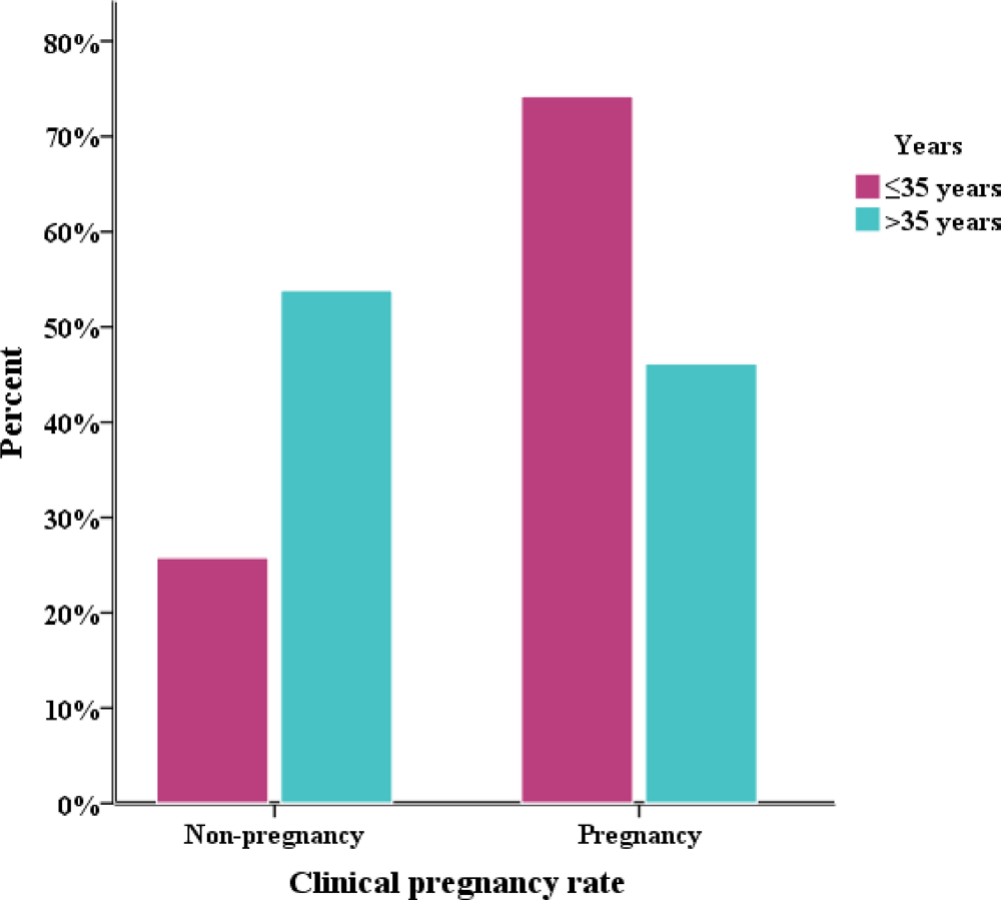
Comparison between years and clinical pregnancy.

Table 4 shows the comparison of results obtained between patients who did not have confirmed pregnancies (the group A) and patients with confirmed pregnancies (the group B) (Figs. 2 and 3). A statistically significant difference (*P* > .05) between 2 groups of patients was confirmed for the age (*P* = .034), serum AMH concentration (*P* = .005), the number of oocytes (*P* = .000), the number of mature MII cells (*P* = .000), the number of embryos (*P* = .000) and the concentration of IL-6 in the follicular fluid (*P* = .000).

**Table 4:** Comparison between nonpregnancy and pregnancy groups for clinical characteristics.

**Parameter**		Group A (n = 36)No pregnancy	Group B (n = 47)confirmed pregnancies	*P* value
**Age (yr**)		38.14 ± 4.72	36.02 ± 4.20	**0.034** *
**BMI**		27.58 ± 1.87	27.04 ± 1.92	0.206†
**AMH in serum (ng/mL**)		1.78 ± 2.09	2.74 ± 2.16	**0.005** †
**Oocytes retrieved**		5.44 ± 3.54	9.45 ± 4.88	**0.000** †
**MII oocytes**		3.14 ± 2.03	5.94 ± 3.14	**0.000** †
**Methods (%**)	IVF	11.1	88.9	0.113‡
	ICSI	46.6	53.7	
	IVF/ ICSI	50	50	
**Embryos**		2.69 ± 1.98	4.87 ± 2.87	**0.000** †
**FF IL-6 on oocyte retrieval day (ng/mL**)		2.70 ± 0.59	9.55 ± 7.47	**0.000** †

The values are given as mean ± standard deviation. Statistically significant *P* values are given in bold.

AMH = antiMullerian hormone, BMI = body mass index, FF = follicular fluid concentrations, Group A = cycles that did not result in pregnancy, Group B = cycles that resulted in pregnancy, IVF = in vitro fertilization, ICSI = intracytoplasmic sperm injection.

* *P* value estimated by Independent Sample *T* test.

† *P* value estimated by Mann–Whitney *U* test.

‡ *P* value estimated by Pearson chi-square test.

Patients from the group A were older (38.14 ± 4.72) and had lower serum AMH levels (1.78 ± 2.09 ng/mL) compared to patients from the group B (years 36.02 ± 4.20; serum AMH 2.74 ± 2.16 ng/mL). Patients with confirmed pregnancy (the group B) had more oocytes (9.45 ± 4.88), more MII cells (5.94 ± 3.14), as well as embryos (4.87 ± 2.87) in relation to patients in whom pregnancy was not confirmed—the group A (oocytes 5.44 ± 3.54; M II cells 3.14 ± 2.03; embryos 2.69 ± 1.98). The concentration of IL-6 in the follicular fluid was higher in patients with confirmed pregnancy (9.55 ± 7.47 ng/mL).

Table 5 shows the correlation between the concentration of IL-6 in the follicular fluid and the parameters monitored during this study. The study showed a statistically significant correlation with a positive coefficient between IL-6 concentration in the follicular fluid and serum AMH concentration (*rho* = 0.289; *P* = .008; weak bond), IL-6 concentration in the follicular fluid and the number of obtained oocytes (*rho* = 0.348; *P* = .001; moderate-strong bond), IL-6 concentration in the follicular fluid, and the number of mature MII cells (*rho* = 0.419; *P* = .000; strong bond), IL-6 concentration in the follicular fluid, and the number of obtained embryos (*R* = 0.422; *P* = .000; strong bond), and IL-6 concentration in the follicular fluid and the outcome of the IVF process (*rho* = 0.834; *P* = .000; strong bond). A positive correlation indicates that higher concentrations of IL-6 in the follicular fluid are followed by higher concentrations of AMH in the serum, a higher number of oocytes/MII cells/embryos, and a positive outcome of the IVF process, and vice versa. A statistically significant correlation with a negative coefficient has been demonstrated in terms of age and IL-6 concentration in the follicular fluid (*rho* = -0.211; *P* = .050, weak bond); therefore, the higher serum IL-6 concentrations in the younger patients were and vice versa.

**Table 5:** Correlations of the clinical characteristics and the concentrations of FF IL-6 (ng/mL).

**Parameter**	r/rho	*P* value
**Age (yr**)	–0.211	0.050
**BMI**	–0.259	0.018
**AMH (ng/mL**)	0.289	0.008
**Оocytesretrieved**	0.348	0.001
**MII oocytes**	0.419	0.000
**Embryos**	0.422	0.000
**Outcome**	0.843	0.000

Statistically significant *P* values are given in bold.

AMH = antiMüllerian hormone, BMI = body mass index, FF = follicular fluid concentrations, IL-6 = Interleukin 6, rho = Spearman correlation coefficient.

**Figure 4.**
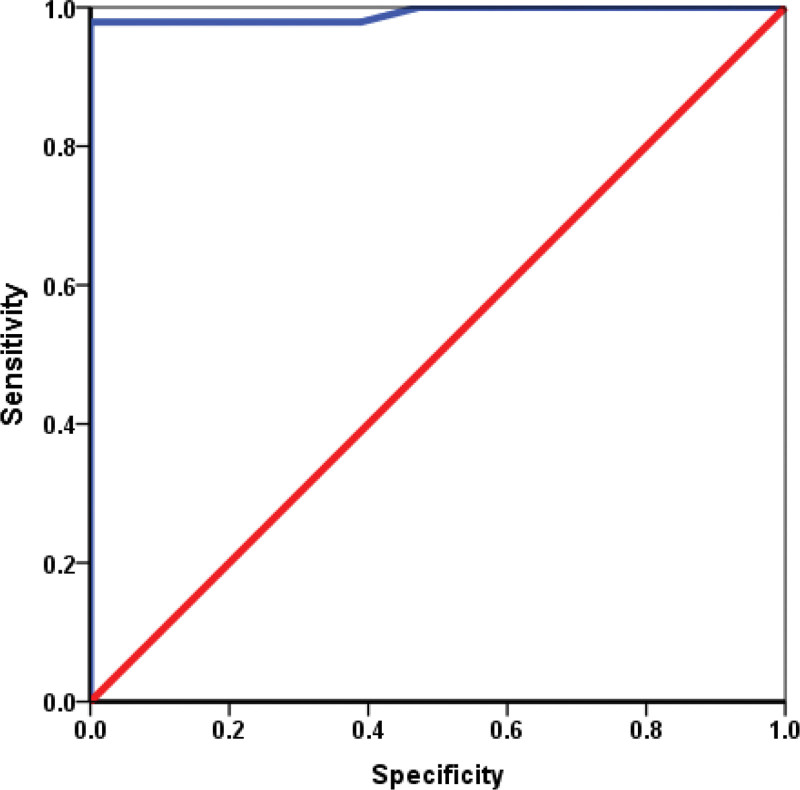
ROC analysis for IL-6 values in follicular fluid as a potential marker of a positive outcome of the IVF process.

Table 6 shows the results of the *ROC* analysis, which we used to determine whether IL-6 from the follicular fluid could be a predictive marker of the outcome of in vitro fertilization. Fig. 4 presents a graphical representation of *ROC* analysis. As shown in Fig 4., the area for IL-6 was highly statistically significant (*P* = .000) and distant from 0.5, and close to 1 (area = 0.991). *The cut-off* value was > 3.67 ng/mL (sensitivity 96.7; specificity 83.3).

**Table 6:** ROC analysis (IL-6 from follicular fluid as predictors of IVF outcome).

	Surface area	*P*	95% confidence interval	
			Lower limit	Upper limit
**IL-6 (ng/mL**)	0.991	**0.000**	0.972	1000

Statistically significant *P* values are given in bold.

IL-6 = Interleukin 6.

## 4. Discussion

Patient age is one of the most important factors associated with in vitro fertilization success. The ability of egg cells to produce high-quality embryos that will lead to healthy pregnancy decreases with patient years.^[25]^ The mean years of the patients included in this study was 36,94.

A positive outcome was noted in younger patients. The mean age of patients with the positive outcome was 36,02 ± 4,20, while the mean age of patients with the negative outcome was 38,14 ± 4,72. Previous research has shown that the optimal age for IVF success is <36 years,^[26]^ which has been proven in this study. As a woman grows older, fertility significantly decreases, as confirmed by Nye et al Approximately 10 % of women cannot get pregnant naturally by the years of 34, while 87% cannot get pregnant naturally between the years of 40 and 45. The success rate of IVF is significantly lower among older women, although IVF may overcome the fertility decline in older women to some extent.^[27]^

Several studies have confirmed that the infertility risk is 3 times higher in obese women than in women with normal weight. Obesity influences egg cell maturation, endometrial development, implantation, and miscarriage.^[28]^ Returning to a normal weight improves not only women’s health but also the rate of positive pregnancy.^[28,29]^ The body mass index was within the normal range (<30 kg/m^2^), which is one of the key criteria for inclusion in the study.

The value of hormone AMH in the serum is important because it shows the ovarian reserve and normal value (1–4 ng/mL), indicating that the ovary still has enough egg cells needed for fertilization. The value of AMH in the serum is a good predictor of ovarian response in the IVF process.^[30]^ Based on the results obtained in our study, the mean value of AMH in the serum per patient was higher in patients who were 35 years old or less, as well as in patients with a positive outcome.

Response to ovarian stimulation varies from patient to patient. A larger number of obtained egg cells increased the success rate of the IVF process. A greater number of egg cells gives more embryos that can be cultivated to the blastocyst stage, enabling the selection of the best quality embryos for transfer.^[31–34]^ In IVF, obtaining good-quality embryos is the most important for successful implantation and positive outcomes.^[32,35]^ Based on previous studies, most studies have concluded that the optimal number of obtained egg cells is between 8 and 18.^[31,36,37]^ In our study, the average number of obtained egg cells per patient was 7.71 and the number of embryos per patient was significantly lower (3.93). By analyzing the quality of the obtained egg cells of the patients included in our study, the number of mature MII egg cells per patient was 4.72.

ICSI is one of the most commonly used techniques of assisted fertilization for infertility treatment in couples with the absence of conception.^[38]^ Considering the applied method of fertilization, that is, whether a conventional IVF, ICSI, or a combination of both methods was used in our study, we determined that the ICSI method was applied most often not only in younger patients but also in older patients. In addition, the ICSI method was most commonly applied in patients with negative or positive outcomes of the IV fertilization process. The difference in the presence of fertilization methods in the groups was not statistically significant. Some previous studies have also concluded that there is no difference between IVF and ICSI in relation to fertilization and the outcome of the process.^[39,40]^ The reason for this excessive use lies in the fact that ICSI has become an acceptable method for the treatment of female infertility in cases where the egg cells are of poor quality or where there are only a few cells as well as in older women because of the frequent absence of a mechanism of locking the cell after the first sperm penetration into the cell.^[41,42]^

The high developmental potential of a good-quality egg cell is closely related to events that occur before follicle formation, as well as during folliculogenesis. Maturation, development, and differentiation of somatic and egg cells within the follicle depend on the regulation of gene expression and enzyme activity, as well as growth factors and nutrients that are locally produced or brought into the follicle.^[43,44]^ Biological processes within the follicle can be modeled and analyzed for molecular identification and signal pathways that influence the quality of egg cells, as well as influences from the environment.^[45–47]^

Follicular fluid is a biological material that can be obtained during in vitro fertilization cycles and is considered as the optimal source of markers for predicting oocyte quality and the success of IVF. The end products of cellular metabolism are low molecular weight metabolites that can be detected as the response of the follicle to all the influences that affect its development.^[48–51]^ We analyzed only 1 molecule (IL-6) related to ovarian immune function and obtained very interesting results for the continuation of this type of study in the future.

In our study, the mean value of IL-6 in the follicular fluid per patient was 6.59 ± 6.29 ng/mL. The results of our study showed that there was a statistically significant difference between the 2 groups of patients that were divided first on the basis of their age (≤35 years and > 35 years), and then, according to the outcome of the IVF process (negative and positive). Higher concentrations were noted in younger patients and patients with a positive outcome of the IVF process. The results of a previous study demonstrated that higher values of IL-6 in the follicular fluid were found in pregnant women (8.39 ± 7.95 pg/mL),^[52]^ which was shown in our study.

Our results indicate that IL-6 concentration is higher in women with lower BMI, which has not been proven in some other studies whose results suggest that IL-6 concentration is higher in obese women, which is also consistent with a decrease in the oocyte quality. IL-6 is an inflammatory cytokine that may have antiinflammatory or pro-inflammatory effects in a variety of physiological states, such as obesity, chronic inflammation, and endometriosis.^[15]^

Our results indicate that IL-6 concentration in the follicular fluid is higher in women with higher AMH concentrations in the serum, as well as in women younger than 35 years, which is related to the outcome of IVF (a higher success rate of in vitro fertilization in women younger than 35 years and in women who have higher AMH).

Our results indicate that there is a positive effect of IL-6 in the follicular fluid on the quality of oocytes and embryos, as well as on the outcome of IVF; higher concentrations of IL-6 (9.55 ± 7.47 ng/mL) have been observed in the follicular fluid of women in whom pregnancy has been confirmed, which is in line with previous studies (pregnancy 8.39 ± 7.95 pg/mL vs non- pregnancy 4.64 ± 2.75 pg/mL).^[52]^ Previous studies have suggested the opposite effect of IL-6. Altun et al showed that higher concentrations of IL-6 (8.39 ± 4.48 pg/mL) in follicular fluid negatively affected the outcome of IVF. Their results also showed that there was no correlation between the number of oocytes obtained and embryo quality. They believe that endometrial receptivity as well as the harmful effect of high IL-6 concentration in the endometrium are more important for the success of IVF.^[17]^

Based on the design of our study, the follicular fluid samples for each patient were pooled, so IL-6 concentrations could not be related to oocytes from each follicle individually, but the correlation was observed between IL-6 concentrations in the follicular fluid and the total number of obtained oocytes, as well as the number of mature oocytes. This was confirmed by a group of authors,^[12]^ who concluded, based on their results, that higher IL-6 concentrations in the follicular fluid correlate with a larger number of mature oocytes obtained from these follicles. IL-6 is a cytokine that participates in follicular development and maturation by inducing the expansion of the cumulus-oocyte complex and by inducing the expression of ovulation-related genes.^[52]^

It has been observed that IL-6 concentrations between 10 ng/mL and 44 ng/mL in the follicular fluid lead to spontaneous abortion during early pregnancy. Of the 83 patients included in the study, 11 had a miscarriage in early pregnancy and their IL-6 concentration in the follicular fluid exceeded 10 ng/mL. In our study, the highest concentration of IL-6 in the follicular fluid was 44 ng/ml, and the outcome of this process was a miscarriage. Earlier research arrived at similar conclusions.^[53]^ This suggests that extremely high IL-6 (>10 ng/mL) concentrations or a lack of IL-6 are associated with IVF failure or pregnancy loss.

Measurement of IL-6 concentration in the follicular fluid as a predictive marker of the positive IVF outcome may be of great importance for the possible introduction of adequate immunosuppressive therapy if concentration is > 10 ng/mL in the follicular fluid. Our results show that if the IL-6 concentration in the follicular fluid is higher than the *cut-off* value (>3.67 ng/mL), in 96.7% of patients with 83.3% specificity, the positive outcome of IVF can be predicted.^[4]^

As a proinflammatory cytokine, IL-6 is produced by the epithelial and stromal cells of the endometrium during implantation, so it is considered that IL-6 induces decidualization of the uterine stroma. IL-6 receptors are located on the endometrium, trophoblasts, and blastocysts. Interleukin 6 is the key to a successful pregnancy, with low levels in the proliferative phase and steady rise during the secretory phase of the menstrual cycle.^[53]^

Contradictory results of studies related to the relationship between the IL-6 levels in the follicular fluid and the outcome of IVF suggest that large studies should be performed with a large number of samples. Most studies are designed to find a good marker that will help assess the quality of oocytes and to increase the success of IVF outcomes, but most have not been done on a highly prospective and well-controlled basis. Consequently, no reliable marker has been identified so far on the basis of which the quality of oocytes can be predicted.^[17,48,54]^

## 5. Conclusion

Infertility is a serious issue today, and despite the fact that medicine has advanced, it is difficult to identify all interactions between various cells that participate in fertilization and nidation processes. Increasing attention is being paid to the composition of the follicular fluid, and this is where the cause of infertility is sought. This study aimed to determine whether interleukin from follicular fluid affects the quality of oocytes, and therefore, the rate of fertilization and IVF outcome. Based on our results, it can be concluded that IL-6 concentration in the follicular fluid affects the quality of oocytes, as well as the fertilization rate and outcome of IVF in a specific way. Our study concluded that if the range of IL-6 concentrations is between 3.67 ng/mL and 10 ng/mL, we can expect good IVF outcome with vital pregnancy. In order to improve further research and to obtain results that will help solve the problem, it would be beneficial for researchers to conduct studies in a similar way to determine the exact range of concentrations and influence of IL-6.

## Author contributions

Conceptualization: Aleksandra Z. Stojanovic Gavrilovic, Marija P. Sorak

Data curation: Aleksandra Z. Stojanovic Gavrilovic, Jelena M. Cekovic, Aida Z. Parandilovic

Formal analysis: Aleksandra Z. Stojanovic Gavrilovic, Aleksandra M. Velickovic, Marija V. Andjelkovic

Investigation: Aleksandra Z. Stojanovic Gavrilovic, Jelena M. Cekovic, Aida Z. Parandilovic

Methodology: Aleksandra Z. Stojanovic Gavrilovic, Aleksandar B. Nikolov, Aleksandra M. Velickovic

Project administration: Aleksandra Z. Stojanovic Gavrilovic, Jelena M. Cekovic

Resources: Aleksandra Z. Stojanovic Gavrilovic, Aida Z. Parandilovic

Software: Aleksandra Z. Stojanovic Gavrilovic, Aleksandar B. Nikolov

Supervision: Marija P. Sorak, Predrag S. Sazdanovic

Validation: Marija P. Sorak, Predrag S. Sazdanovic

Visualization: Aleksandra Z. Stojanovic Gavrilovic, Marija V. Andjelkovic

Writing – original draft: Aleksandra Z. Stojanovic Gavrilovic

Writing – review & editing: Jelena M. Cekovic, Aida Z. Parandilovic, Aleksandar B. Nikolov, Predrag S. Sazdanovic, Aleksandra M. Velickovic, Marija V. Andjelkovic, Marija P. Sorak
